# Effects of Oleic Acid and Intramuscular Fat Levels on Retronasal Aromas in Wagyu Beef from Japanese Black Cattle

**DOI:** 10.3390/foods15060994

**Published:** 2026-03-11

**Authors:** Naoaki Obana, Yuri Yoshida, Kazunori Matsumoto, Masakazu Irie

**Affiliations:** National Livestock Breeding Center, Nishigo-mura 961-8511, Fukushima, Japan; n0obana@nlbc.go.jp (N.O.); y0yoshida@nlbc.go.jp (Y.Y.); k0matsumoto@nlbc.go.jp (K.M.)

**Keywords:** Wagyu beef, intramuscular fat, fatty acid, oleic acid, aroma

## Abstract

This study aimed to evaluate the effects of various levels of intramuscular fat (IMF) and oleic acid (C18:1 cis-9) on retronasal aromas in Wagyu beef. Muscle samples were collected from 167 carcasses of Japanese Black. The chemical compositions were analyzed, and the cooked beef was evaluated by a trained sensory panel. Tenderness, juiciness, and fatty aroma were mainly related to the IMF content. Both sweet and Wagyu beef aromas were affected by the oleic acid and IMF contents. In marbled beef with low IMF content, both sweet and Wagyu beef aromas were stronger as the oleic acid composition increased (r = 0.401, 0.376); however, their relationships were weaker at the moderate IMF content (r = 0.278, 0.273). The effect of oleic acid on these aromas was hardly observed in beef with high IMF content (r = 0.030, 0.011). The oleic acid index [IMF content (%) × oleic acid composition (%)/100] could be fitted to the logarithmic curve for all the aromas determined (r = 0.526 to 0.565). These results indicated that the higher oleic acid composition could be better for the favorable aromas of Wagyu beef; however, the effect differs depending on the IMF content levels, and the phenomenon is relatively well explained by the oleic acid index.

## 1. Introduction

Fat quality is obviously related to the eating quality of beef. First, fatty acids (FAs) influence the melting point of fat [1]. In particular, Japanese Black cattle (JB) beef contains a high level of oleic acid (C18: 1 cis-9) and exhibits a very low melting point, so the melted fat has a favorable effect on the texture containing juiciness and melt-in-the-mouth feeling [2].

Second, fat also affects beef flavors. Triacylglycerol, which makes up the majority of fats, is a large molecule and nonvolatile, cannot act on the receptors in gustatory and olfactory organs, and is tasteless and odorless. However, when some of the triacylglycerol is hydrolyzed into free fatty acids (FFAs), free oleic acid and free linoleic acid impart a unique taste (fat taste) on the gustatory organ [3,4]. Fat taste is attracting attention not only as a candidate for the sixth basic taste [5] but also as a factor that affects health through appetite regulation, and many studies are currently being conducted [6].

Fats with a low melting point are more likely to release odorous fat-soluble components. In addition, FFAs are decomposed into volatile flavor compounds through various oxidation mechanisms [7,8]. In particular, JB beef has a unique sweet and fatty aroma (Wagyu beef aroma), which is thought to be mainly related to lactones [9,10]. Lactones are thought to be derived from FAs [7,11,12]. Among the FAs, monounsaturated fatty acids (MUFAs) such as oleic acid are likely to relate to the favorable aroma of beef [2,7,13,14,15,16,17].

Therefore, not only intramuscular fat (IMF) content but also FA and FFA contents are important for flavor. However, they are difficult to quantify because their analysis takes time and costs, and FFAs are produced in a short time by storage and heating, becoming volatile compounds as a result of chemical changes. Therefore, the FA index [IMF content (%) × FA composition (%)/100] [2], which reflects the amount of FAs, was used in this study. The FA index can also be an indicator reflecting the FFA content if there is no significant difference in the release rate of FAs in a fixed condition. However, the validity of the FA index for flavor has not yet been surveyed. Therefore, this study aimed to investigate the effects of IMF content, FA composition such as oleic acid, and FA index on the aroma of JB beef.

## 2. Materials and Methods

### 2.1. Sample Collection

Loins from the 7th to the 10th ribs were obtained from 167 JB carcasses at eight meat markets in Japan. All 167 JBs were steers and had a slaughter age of 28.1 ± 1.6 months. The loins were stored in the refrigerator (IMS-1198; AS ONE Corporation, Osaka, Japan) at 2 °C. The samples of the *Longissimus thoracis* muscle (*LT*) were collected 14 days after slaughter, vacuum-packed and frozen at −30 °C.

### 2.2. Chemical Analysis

The moisture, IMF, and crude protein contents were analyzed twice per sample based on the procedure described by Okumura et al. (2012) [18]. The moisture content was determined by heating 2.0 g of the minced *LT* sample at 105 °C for 24 h and calculating the difference in weights before and after heating. The sample obtained after measuring the moisture content was extracted for 16 h using diethyl ether (FUJIFILM Wako Pure Chemical Corporation, Osaka, Japan) to extract the fat, and the IMF content was determined. The crude protein content was determined using the Kjeldahl method for 1.0 g of minced *LT* samples.

The FA composition was analyzed twice per sample based on the procedure described by Sakuma et al. (2017) [19]. IMF lipids were extracted from *LT* samples frozen about 30 days, with a chloroform (NACALAI TESQUE, Inc., Kyoto, Japan)/methanol (FUJIFILM Wako Pure Chemical Corporation, Osaka, Japan) ratio of 2:1, methylated with 0.5 N sodium methoxide methanol (KANTO CHEMICAL Co., Inc., Tokyo, Japan), and then extracted to n-hexane (FUJIFILM Wako Pure Chemical Corporation, Osaka, Japan). FA methyl esters were analyzed by a gas chromatograph (6890A; Agilent Technologies Japan, Tokyo, Japan) equipped with a flame ionization detector (FID) and a capillary column (TC-70, 0.25 mm × 60 m; GL Science, Tokyo, Japan). Oven temperature was held at 190 °C for 8 min following injection, then increased to 230 °C at a rate of 20 °C per min and held at 230 °C for 2 min. The carrier gas was helium at a flow rate of 1.8 mL per min. The following sixteen FAs were identified: lauric acid (C12:0), myristic acid (C14:0), myristoleic acid (C14:1), pentadecylic acid (C15:0), palmitic acid (C16:0), palmitoleic acid (C16:1), margaric acid (C17:0), heptadecenoic acid (C17:1), stearic acid (C18:0), elaidic acid (C18:1 trans-9), oleic acid (C18:1 cis-9), linoleic acid (C18:2), linolenic acid (C18:3), arachidic acid (C20:0), eicosenoic acid (C20:1), and arachidonic acid (C20:4), each from the retention time of each standard material (GL Science, Tokyo, Japan). Each FA composition (%) was expressed as a percentage of the total of all sixteen FAs, and saturated fatty acids (SFA = C12:0 + C14:0 + C15:0 + C16:0 + C17:0 + C18:0 + C20:0), MUFAs (MUFA = C14:1 + C16:1 + C17:1 + C18:1 trans-9 + C18:1 cis-9 + C20:1), and polyunsaturated fatty acids (PUFA = C18:2 + C18:3 + C20:4) were calculated. In addition, since the FA composition was relative values, quantitative FA indices [IMF content (%) × FA composition (%)/100) were calculated.

### 2.3. Sensory Evaluation

#### 2.3.1. Preparation of Beef Samples

Vacuum-packed *LT* samples frozen on the 14th day after slaughter were thawed in the refrigerator (MPR-514-PJ; Panasonic Healthcare Co., Ltd., Tokyo, Japan) at 2 °C for 24 h, and heated to the internal temperature of 70 °C in a drying oven (DS401; Yamato Scientific Co., Ltd., Tokyo, Japan) at 165 °C [20]. Internal temperature in *LT* samples was monitored with copper–constantan thermocouples (JT6; CHINO, Tokyo, Japan) and a digital thermometer (MC1000; CHINO, Tokyo, Japan). After heating, the samples were left to cool at room temperature (24 °C) for 10 min, cut into pieces of 3 × 3 × 0.5 cm, placed in a stainless-steel container with lids and stored in a convection oven (DG800; Yamato Scientific Co., Ltd., Tokyo, Japan) at 40 °C. Four samples were served to the panelists per JB.

#### 2.3.2. Evaluation of Beef Samples

The panelists were trained in the evaluation terminology and evaluation scales of beef under the guidance of a panel leader beforehand. In the evaluation training, the scales were adjusted on scales of 1–6 (1 = extremely tough, extremely dry, and extremely weak; 6 = slightly tough, slightly dry, and slightly weak) and 7–12 (7 = slightly tender, slightly juicy, and slightly strong; 12 = extremely tender, extremely juicy, and extremely strong).

The sensory evaluation was performed in multiple sessions and evaluated by 30 in-house panelists who passed the odor and taste discrimination test [20]. A total of 3–7 panelists (average of 4.8 panelists) evaluated a sample of 2-3 JB in one session. A Latin square design was used to avoid effects of serving order.

The panelists evaluated the tenderness, juiciness, fatty, sweet, and Wagyu beef aromas of the served samples on a scale of 1–12 (1 = extremely tough, extremely dry, and extremely weak; 12 = extremely tender, extremely juicy, and extremely strong). The fatty aroma was evaluated as a butter-like aroma; the sweet aroma as a peach-like, coconut-like, and boiled corn-like aroma; and the Wagyu beef aroma as a sweet and fatty aroma. Tenderness and juiciness ware evaluated as follows: with the nostrils pinched, the sample was placed in the mouth and chewed ten times. The retronasal aroma of these aromas was evaluated as follows: with the nostrils pinched, the sample was placed in the mouth and chewed five times; afterward, the nasal passages were opened, and the sample was chewed an additional 20 times. This evaluation was performed in individual booths within an exclusive room, illuminated by red light to minimize the effect of the sample’s appearance.

### 2.4. Statistical Analysis

Simple and multiple regression analyses were performed to determine whether the fat content, FA composition, and FA index were related to a favorable flavor. In the multiple regression analysis, the objective variable was the sensory evaluation values, and the explanatory variables were the IMF content and sixteen fatty acid indices or IMF content and indices of SFAs, MUFAs, and PUFAs. The stepwise method was applied to the multiple regression analysis, and the baseline for the adoption judgment was *p* = 0.05.

In addition, based on the Weber–Fechner law [21] on flavor, the effects of oleic acid content and oleic acid index on sensory values were calculated by logarithmic curve regression. All statistical analysis was performed by JMP software version 11 (SAS Japan, Tokyo, Japan).

## 3. Results

### 3.1. Descriptive Statistics of Beef

The descriptive statistics of the marbling score and chemical analysis values of the JB beef are shown in Table 1. The beef marbling standard (BMS) of beef carcass grading in Japan (1–12) in this study was widely distributed, ranging from 3 to 12. The chemical contents were also widely distributed. Although the data are not presented in a table, very strong negative correlations were found between the IMF content and crude protein content or moisture content (r = −0.98 and r = −0.99, respectively; *p* < 0.01).

The mean of oleic acid composition was about half the proportion of all FAs; however, differences of up to 16% were observed between individuals. The mean of oleic acid composition occupied almost 90% of MUFAs.

**Figure 1:**
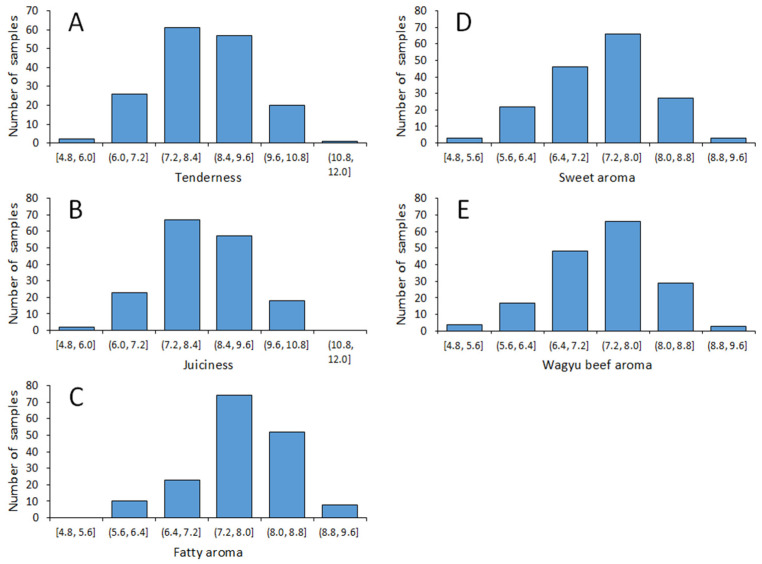
Distribution of sensory evaluation properties for *M. longissimus thoracis* of JB: tenderness (**A**), juiciness (**B**), fatty aroma (**C**), sweet aroma (**D**), and Wagyu beef aroma (**E**). *n* = 167.

Figure 1 shows the distribution of the evaluation values by the analytical sensory panel test in JB beef. Although the ranges of the aroma values were not wide, some individual differences were observed.

### 3.2. Correlation with Beef Aroma

Table 2 shows the relationships between BMS or chemical analysis values and sensory evaluation values in JB beef. The BMS and IMF content demonstrated moderate positive correlations with each sensory evaluation item (*p* < 0.01). The moisture and crude protein contents exhibited moderate negative correlations with their items (*p* < 0.01).

Tenderness, juiciness, and fatty, sweet, and Wagyu beef aromas had a weak or little correlation with almost all FA compositions, with the exception of arachidonic acid. On the other hand, the FA indices showed a weak or moderately positive correlation with each sensory evaluation item, except for some FA indices such as the arachidonic acid index. Among them, the oleic acid index and MUFA index demonstrated the strongest positive correlation with the sweet and Wagyu beef aromas (*p* < 0.01).

### 3.3. Multiple Regression Analysis of Beef Aroma

Table 3 shows the results of analyzing whether the most important factor for these aromas was the IMF content or the FA index by multiple regression analysis. When the IMF content and the FA indices were used as explanatory variables (Analysis 1), the IMF-only equation (No. 1) was adopted for the fatty aroma.

The same was also true when the IMF content, SFA, MUFA, and PUFA indices were used as explanatory variables in Analysis 2 (No. 4). On the other hand, similar coefficients of determination were obtained when the oleic acid index or the MUFA index was used as an explanatory variable instead of the IMF content for the fatty aroma (No. 1′ or 4′).

For sweet aroma, only the oleic acid index was selected in Analysis 1 (No. 2). A similar result was obtained for the MUFA index in Analysis 2 (No. 5).

For the Wagyu beef aroma, the oleic acid and pentadecylic acid indices were selected as the first equation (No. 3). The oleic acid index contributed considerably more to Wagyu beef aroma than the pentadecylic acid index. On the other hand, a similar coefficient of determination was obtained when only the oleic acid index was used as the explanatory variable (No. 3′). Only the MUFA index was selected in Analysis 2 (No. 6).

### 3.4. Relationship Between Beef Aroma and Fat Quality

In the above, significant relationships were found between aromas and chemical components as a linear regression; however, the amount of substances for odor follows a quadratic curve (Weber–Fechner law) [21]. The FFA content increases mainly during the aging period by releasing them from triacylglycerol, and aging improves the flavor of JB [22]. Therefore, the content of FFAs used as raw materials for lipid-derived volatile compounds differs depending on the IMF level. As a result, the content of the volatile compounds produced should also be different.

**Figure 2:**
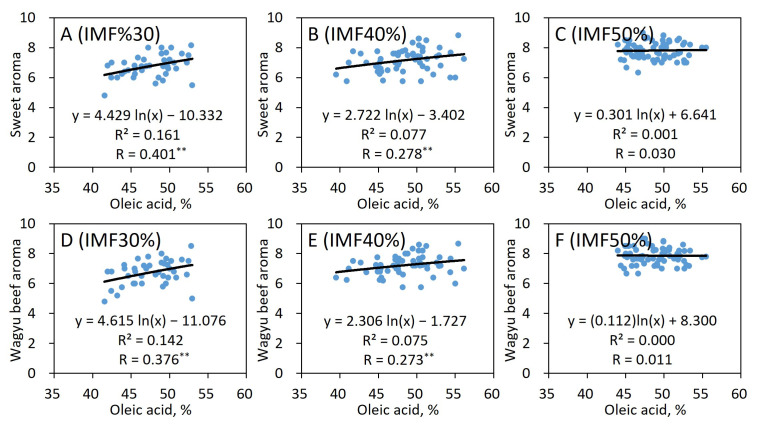
Relationships between aroma and oleic acid composition in *M. longissimus thoracis* of JB with IMF content in the 30% level (**A**,**D**), 40% level (**B**,**E**), and 50% level (**C**,**F**). *n* = 167, ** *p* < 0.01.

Therefore, Figure 2 shows the relationships between the oleic acid composition and sensory evaluation values of aromas of JB beef with IMF levels of 30%, 40%, and 50%. Both the sweet and Wagyu beef aromas became stronger at the 30% IMF level (*p* < 0.01) with an increase in the oleic acid composition, and the slope of the quadratic curve became moderated at the 40% IMF level (*p* < 0.01). At the 50% IMF level, little effect of the oleic acid content was noted on these aromas, and these relative curves remained flat.

**Figure 3:**
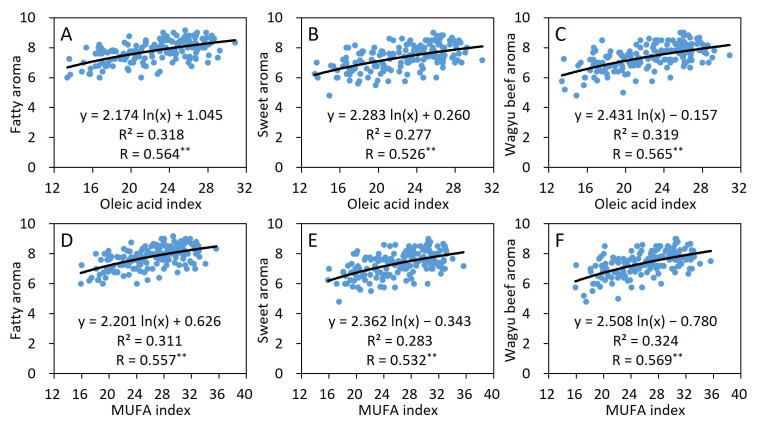
Relationships between aroma and the oleic acid index (**A**–**C**) or MUFA index (**D**–**F**) in *M. longissimus thoracis* of JB. *n* = 167. ** *p* < 0.01.

Given that analyzing only the relationship between the relative level of FA composition and the flavor of the beef with varying IMF content is inappropriate, the idea that the FA index reflects the FA content has been proposed [2]. Therefore, the relationship between the aroma of JB beef and the oleic acid index or MUFA index was shown using logarithmic regressions (Figure 3). The oleic acid index incorporated the FA and IMF contents, and can be shown in a single graph more easily compared with Figure 2. For example, Figure 2D–F corresponds to Figure 3C. A moderately significant correlation of approximately 0.5 was obtained for these aromas. This was also the case with the MUFA index.

## 4. Discussion

### 4.1. Beef Characteristics

The results of this study indicate that IMF content and fat quality vary significantly between JB individuals (Table 1). Following the liberalization of beef imports in Japan in 1991, genetic improvement with an emphasis on marbling has been promoted in JB to differentiate it from imported beef [23]. Because marbling is one of the most important factors in determining the grade of meat quality, JB marbling has increased annually, and the mean BMS number in JB slaughtered in 2022 was >8.0 [24]. The increase in IMF content was mainly due to the genetic improvement of BMS in JB, which is related to eating quality attributes such as texture (tenderness and juiciness) and flavor [2,25,26]. Fat quality was also identified as being related to eating quality and nutrition, and it is considered a genetic factor separate from BMS [2].

The large variation was confirmed between individuals even in the oleic acid composition. Compared with other breeds, JB have higher compositions of oleic acid and MUFAs, and a lower melting point of fat [2,27,28]. However, in JB, the focus so far has been on the genetic improvement of marbling; thus, little genetic improvement in FAs has been done. This is one of the reasons for the large variations in FAs between individuals in JB.

### 4.2. Factors Affecting Beef Aroma

The IMF content exerted a stronger influence on the fatty aroma than on the FA composition and FA index. Similarly, Mu et al. (2023) [29] reported that meat with different fat content can be distinguished by smell. The positive correlation identified between the fatty aroma and many SFA, MUFA, and PUFA indices (Table 2) can be explained by the fact that the FA index is influenced by IMF content. This also applies to sweet and Wagyu beef aromas, but the oleic acid and MUFA indices were more strongly related to these aromas than IMF content and the other FA indices. Thus, not only IMF content but also fat quality is important for sweet and Wagyu beef aromas.

These reasons include the formation of flavor compounds from FAs. Some FAs present in lipids are released by hydrolysis, and flavors are produced by further chemical changes. That is, FFAs are produced from FAs by lipase, heating, etc. Then, FFAs undergo oxidation through various mechanisms to form hydroperoxides, which ultimately produce volatile compounds such as aldehydes, ketones, and esters, which are important for the formation of meat flavor [7,8]. In particular, it has been suggested that lactones with a sweet aroma may be made from oleic acid and linoleic acid [7,11,12,30]. In addition, lactones may be important substances in the Wagyu beef aroma [9,11,31].

Thus, the strong association between the sweet or Wagyu beef aromas and the oleic acid or MUFA indices in this study indicates that lactones and other favorable aroma compounds are likely to be produced from free oleic acid (Table 2 and Table 3 and Figure 3). In another experiment (*n* = 15), the analysis of the relationship between the oleic acid index and the free oleic acid content revealed a strong correlation, with r = 0.74 (*p* < 0.01). In addition, the odor of lactones alone was not equal to the sweet aroma of JB beef, and it did not show such a strong correlation with the sweet aroma in the sensory evaluation (unpublished). Therefore, other aroma compounds produced from oleic acid can also affect flavor, and the fat, sweet, and umami tastes by free oleic acid may also affect the retronasal aroma through flavor acceptance.

### 4.3. Effect of FAs on the Beef Aroma

Fat quality is important because the effect of the oleic acid composition on the beef aroma is influenced by the IMF level (Figure 2). In addition, since the intensity of the sweet and Wagyu beef aromas did not decrease with the increase in the oleic acid composition at any IMF content level, the high oleic acid composition did not negatively affect these aromas at the level of up to approximately 60%, as used in this study.

These results are consistent with the view that high MUFA levels, such as oleic acid, affect the preferred flavor of beef [2,7]. The preferred aroma of beef is possibly related to the difference in the content of free oleic acid, which can be used as a raw material for these volatile compounds. That is, beef with low IMF content has a low content of FAs such as oleic acid, which is a raw material for volatile compounds such as FFAs. In contrast, beef with high IMF content contains high concentrations of oleic acid and their derivatives as beef flavor. Thus, the high oleic acid composition should be conspicuously useful for an increase in the favorable flavor of Wagyu beef with low IMF content (30%). The influence of oleic acid on flavor was weaker in beef with medium IMF content (40%). Conversely, beef with high IMF content (50%) would be supplied with sufficient content of FFAs, and many favorable aroma compounds would be produced. According to the Weber–Fechner law of odor perception, the aroma is thought to little improve above a certain level, which was consistent with the results of this study. In assessing beef flavor, evaluating the flavor alone by the FA composition or IMF content appears inadequate; thus, incorporating the FA index is more appropriate.

Although the range of aroma values was narrow in this study (Figure 1) and beef contains various odor compounds, the oleic acid index and the MUFA index were relatively good standard values of favorable aromas (Figure 3). In Japan, portable near-infrared fiberoptic (NIR) devices for evaluating the fat quality of meat have been developed [32], and a system that can quickly and nondestructively measure the FA composition of JB beef and pork carcasses has been established. In addition, the NIR device can measure easily and quickly the IMF content of *LT* on the carcass line, so that their indices can be obtained rapidly at the carcass stage. The NIR device measures oleic acid, SFA, and MUFA compositions based on seven FAs (myristic acid, myristoleic acid, palmitic acid, palmitoleic acid, stearic acid, oleic acid, and linoleic acid). The correlations between these seven FA compositions identified using the NIR device at the meat market and the sixteen FA compositions identified in this study were all greater than or equal to 0.99. Therefore, these indices obtained from the NIR device may contribute to the evaluation of the eating quality of beef and genetic improvement.

The IMF content in JB has been increasing annually, and beef with an IMF content of >50% has sometimes been seen in recent years. A study also reported a quadratic relationship between the IMF content and the overall sensory evaluation, and the evaluation of beef with an IMF content of approximately 40% was high [25,33]. Therefore, the higher IMF content does not mean that the eating quality will continue to improve in JB beef. In this study, the FA compositions contributed little to the aroma in beef with an IMF content of >50% (Figure 2). Therefore, increasing the FA composition, such as oleic acid, while keeping the IMF content at a level of ≤50%, is important to improve the eating quality of highly marbled beef such as JB.

## 5. Conclusions

The fatty aroma of JB beef was mainly due to the high IMF content. The sweet and Wagyu beef aromas were closely related to the oleic acid index and the MUFA index, which reflect the contents of oleic acid, MUFAs, and their FFAs, rather than the IMF content or FA composition.

The oleic acid index and the MUFA index can be obtained quickly at the carcass stage by optical methods and will be useful as indicators of beef distribution prices and genetic improvement. Given that the IMF content in JB is already at a sufficiently high level in Japan, enhancing the MUFA composition, particularly oleic acid, will be increasingly important as an indicator for improving the eating quality of JB beef.

## Figures and Tables

**Table 1:** Descriptive statistics of the beef marbling standard, chemical composition, fatty acid content, and their indices in the *M. longissimus thoracis* of JB.

Trait	Mean	SD	Minimum	Maximum
BMS	8.1	2.3	3	12
Chemical composition, %				
Moisture	41.0	5.4	31.5	52.6
IMF	46.5	7.3	30.5	59.2
Crude protein	12.3	1.8	8.9	16.4
FA composition, %				
C12:0	0.06	0.01	0.03	0.10
C14:0	2.75	0.54	1.75	4.30
C14:1	1.04	0.26	0.46	1.83
C15:0	0.32	0.07	0.20	0.57
C16:0	25.76	2.23	20.67	32.52
C16:1	4.04	0.67	2.27	6.80
C17:0	0.87	0.17	0.53	1.49
C17:1	1.00	0.16	0.63	1.41
C18:0	11.37	1.75	7.79	19.01
C18:1 trans-9	1.40	0.48	0.68	3.28
C18:1 cis-9	48.36	3.28	39.50	56.14
C18:2	2.29	0.55	1.13	3.88
C18:3	0.13	0.04	0.07	0.28
C20:0	0.07	0.01	0.04	0.11
C20:1	0.44	0.13	0.18	0.94
C20:4	0.09	0.02	0.05	0.18
SFAs ^a^	41.20	3.69	33.64	52.59
MUFAs ^b^	56.29	3.56	45.71	63.98
PUFAs ^c^	2.51	0.59	1.28	4.25
FA index ^d^				
C12:0 index	0.03	0.01	0.01	0.04
C14:0 index	1.27	0.30	0.65	2.15
C14:1 index	0.48	0.15	0.20	0.93
C15:0 index	0.15	0.04	0.07	0.28
C16:0 index	11.95	1.95	7.92	16.00
C16:1 index	1.87	0.39	1.00	3.07
C17:0 index	0.41	0.10	0.19	0.82
C17:1 index	0.46	0.11	0.26	0.76
C18:0 index	5.30	1.21	2.91	8.92
C18:1 trans-9 index	0.65	0.26	0.28	1.69
C18:1 cis-9 index	22.52	3.97	13.36	30.82
C18:2 index	1.07	0.33	0.52	2.13
C18:3 index	0.06	0.02	0.03	0.15
C20:0 index	0.03	0.01	0.02	0.05
C20:1 index	0.21	0.07	0.09	0.45
C20:4 index	0.04	0.01	0.03	0.06
SFA index	19.13	3.29	12.14	26.08
MUFA index	26.20	4.53	15.89	35.61
PUFA index	1.17	0.35	0.58	2.31

*n* = 167. ^a^ SFAs: Saturated fatty acids (sum of C12:0, C14:0, C15:0, C16:0, C17:0, C18:0, and C20:0). ^b^ MUFAs: Monounsaturated fatty acids (sum of C14:1, C16:1, C17:1, C18:1 trans-9, C18:1 cis-9, and C20:1). ^c^ PUFAs: Polyunsaturated fatty acids (sum of C18:2, C18:3, and C20:4). ^d^ FA index = IMF content (%) × FA composition (%)/100. JB: Japanese black cattle; SD: Standard deviation; BMS: Beef marbling standard (1 to 12); IMF: Intramuscular fat; FA: Fatty acid.

**Table 2:** Correlation coefficients between sensory evaluation values and beef marbling standard or analysis values in *M. longissimus thoracis* of JB.

Trait	Tenderness		Juiciness		Fatty Aroma		Sweet Aroma		Wagyu Beef Aroma	
BMS	0.66	**	0.68	**	0.50	**	0.45	**	0.49	**
Moisture	−0.69	**	−0.73	**	−0.57	**	−0.49	**	−0.54	**
IMF	0.69	**	0.72	**	0.58	**	0.49	**	0.54	**
Crude protein	−0.70	**	−0.73	**	−0.58	**	−0.49	**	−0.52	**
C12:0	−0.05		−0.10		−0.20	**	−0.20	**	−0.18	*
C14:0	−0.05		−0.10		−0.19	*	−0.21	**	−0.18	*
C14:1	0.02		0.04		−0.08		0.02		0.02	
C15:0	0.15	*	0.10		−0.06		−0.04		0.00	
C16:0	−0.07		−0.15	*	−0.21	**	−0.32	**	−0.29	**
C16:1	−0.16	*	−0.14		−0.19	*	−0.06		−0.09	
C17:0	0.18	*	0.14		0.07		0.05		0.07	
C17:1	0.10		0.13		0.08		0.18	*	0.20	**
C18:0	0.09		0.05		0.08		−0.08		−0.08	
C18:1 trans-9	0.15		0.10		0.04		0.13		0.10	
C18:1 cis-9	−0.03		0.07		0.16	*	0.26	**	0.24	**
C18:2	0.15		0.11		0.03		0.12		0.08	
C18:3	0.14		0.12		0.09		0.07		0.07	
C20:0	0.00		0.00		0.05		−0.14		−0.10	
C20:1	−0.04		0.05		0.13		0.12		0.16	*
C20:4	−0.42	**	−0.46	**	−0.44	**	−0.41	**	−0.48	**
SFAs	0.01		−0.08		−0.11		−0.27	**	−0.24	**
MUFAs	−0.03		0.06		0.11		0.26	**	0.24	**
PUFAs	0.13		0.10		0.02		0.10		0.06	
C12:0 index	0.36	**	0.34	**	0.17	*	0.13		0.17	*
C14:0 index	0.42	**	0.41	**	0.24	**	0.17	*	0.23	**
C14:1 index	0.38	**	0.42	**	0.23	**	0.28	**	0.30	**
C15:0 index	0.54	**	0.52	**	0.30	**	0.26	**	0.32	**
C16:0 index	0.63	**	0.62	**	0.46	**	0.32	**	0.38	**
C16:1 index	0.38	**	0.43	**	0.27	**	0.33	**	0.35	**
C17:0 index	0.56	**	0.54	**	0.41	**	0.32	**	0.38	**
C17:1 index	0.53	**	0.57	**	0.42	**	0.44	**	0.48	**
C18:0 index	0.53	**	0.52	**	0.46	**	0.29	**	0.32	**
C18:1 trans-9 index	0.40	**	0.37	**	0.27	**	0.30	**	0.30	**
C18:1 cis-9 index	0.59	**	0.66	**	0.56	**	0.52	**	0.56	**
C18:2 index	0.47	**	0.46	**	0.32	**	0.34	**	0.34	**
C18:3 index	0.39	**	0.38	**	0.30	**	0.25	**	0.27	**
C20:0 index	0.44	**	0.47	**	0.41	**	0.23	**	0.27	**
C20:1 index	0.27	**	0.36	**	0.34	**	0.30	**	0.35	**
C20:4 index	0.08		0.05		−0.01		−0.09		−0.14	
SFA index	0.63	**	0.63	**	0.48	**	0.32	**	0.38	**
MUFA index	0.60	**	0.67	**	0.55	**	0.53	**	0.56	**
PUFA index	0.47	**	0.46	**	0.32	**	0.34	**	0.33	**

*n* = 167. * *p* < 0.05; ** *p* < 0.01.

**Table 3:** Results of multiple regression analysis to evaluate the effects of chemical variables on the aroma in *M. longissimus thoracis* of JB.

Objective Variable	No.	Explanatory Variable	R^2^	Adjusted R^2^	RMSE
Analysis 1 ^a^					
Fatty aroma	1 1′	IMF *** C18:1 cis-9 index *** ^c,e^	0.33 0.31	0.33 0.30	0.59 0.60
Sweet aroma	2	C18:1 cis-9 index ***	0.27	0.27	0.69
Wagyu beef aroma	3 3′	C18:1 cis-9 index *** and C15:0 index * ^d^ C18:1 cis-9 index *** ^e^	0.33 0.31	0.32 0.30	0.66 0.67
Analysis 2 ^b^					
Fatty aroma	4 4′	IMF *** MUFA index *** ^e^	0.33 0.30	0.33 0.30	0.59 0.60
Sweet aroma	5	MUFA index ***	0.28	0.27	0.69
Wagyu beef aroma	6	MUFA index ***	0.31	0.31	0.67

*n* = 167. * *p* < 0.05, *** *p* < 0.001. ^a^ Analysis 1 used IMF content and indices of C12:0, C14:0, C14:1, C15:0, C16:0, C16:1, C17:0, C17:1, C18:0, C18:1 trans-9, C18:1 cis-9, C18:2, C18:3, C20:0, C20:1, and C20:4 as explanatory variables. ^b^ Analysis 2 used IMF content and indices of SFAs, MUFAs, and PUFAs as explanatory variables. ^c^ FA index = IMF content (%) × FA composition (%)/100. ^d^ Partial contributions for C18:1 cis-9 index and C15:0 index were 0.28 and 0.05, respectively. ^e^ The case of C18:1 cis-9 index or MUFA index manually selected. RMSE: Residual measurement standard error.

## Data Availability

The original contributions presented in this study are included in the article. Further inquiries can be directed to the corresponding author.

## Abbreviations

The following abbreviations are used in this manuscript:

BMS	Beef marbling standard
FA	Fatty acid
FFA	Free fatty acid
IMF	Intramuscular fat
JB	Japanese black cattle
*LT*	*Longissimus thoracis* muscle
MUFA	Monounsaturated fatty acid
PUFA	Polyunsaturated fatty acid
RMSE	Residual measurement standard error
SD	Standard deviation
SFA	Saturated fatty acid
